# A Case of Allergic Bronchopulmonary Aspergillosis With Failure of Benralizumab and Response to Dupilumab

**DOI:** 10.7759/cureus.42464

**Published:** 2023-07-25

**Authors:** Yasuaki Kotetsu, Hiroaki Ogata, Kachi Sha, Atsushi Moriwaki, Makoto Yoshida

**Affiliations:** 1 Department of Respiratory Medicine, National Hospital Organization Fukuoka National Hospital, Fukuoka, JPN

**Keywords:** interleukin-13, mucus plug, dupilumab, benralizumab, allergic bronchopulmonary aspergillosis

## Abstract

We report a case of a 68-year-old woman who was being treated for bronchial asthma and developed allergic bronchopulmonary aspergillosis (ABPA) that was unresponsive to benralizumab therapy but went into remission with dupilumab therapy. The patient presented with an exacerbation of dry cough and was diagnosed with ABPA based on new diagnostic criteria. Despite the attempted therapeutic intervention, the patient declined to use systemic corticosteroids due to concerns about potential side effects. Subsequently, itraconazole and benralizumab were administered, with temporary relief before relapse. Given the patient's refusal to continue itraconazole and benralizumab, dupilumab was administered as an alternative therapy, which resulted in significant improvement of both symptoms and imaging. Although the use of biological agents for ABPA lacks clear evidence, our results suggest that dupilumab may provide an effective therapeutic strategy.

## Introduction

Allergic bronchopulmonary aspergillosis (ABPA) is a pulmonary disease characterized by hypersensitivity to Aspergillus spp. Dendritic cells in the airways presenting Aspergillus antigen prime naïve T cells, leading to a Th2 CD4+ T-cell response with the secretion of type 2 cytokines such as interleukin (IL)-4, IL-5, and IL-13. This T-cell response promotes inflammatory cell infiltration, including neutrophils and eosinophils, and the synthesis of Aspergillus-specific immunoglobulin E (IgE) [1]. Diagnostic criteria for ABPA include asthma symptoms, blood test (eosinophilia, elevated IgE, and precipitating antibody or IgG for filamentous fungi), immediate cutaneous hypersensitivity or IgE for filamentous fungi, a positive fungal culture (sputum or bronchial lavage fluid), and imaging findings (central bronchiectasis, mucus plug, and high attenuation mucus) [2].

The primary strategy for treating ABPA includes anti-inflammatory therapy with systemic corticosteroids and the reduction of fungal load with antifungal agents. However, long-term treatment with systemic corticosteroids can be problematic due to its various adverse effects. Recently, the therapeutic efficacy of biological agents against refractory ABPA, particularly in patients with uncontrolled asthma, has received more attention. We report a case of ABPA remitted with dupilumab after treatment failure with benralizumab. Based on the efficacy observed in the present case, we speculate that dupilumab may provide a new therapeutic strategy for refractory asthma, especially in cases with prominent airway mucus plug formation, although the choice of biological agents for ABPA remains controversial.

## Case presentation

**Figure 1:**
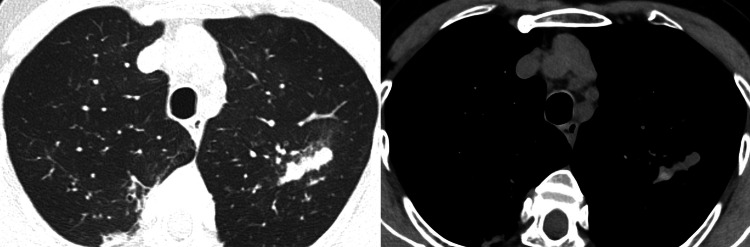
Computed tomography image of the chest when the patient was diagnosed with allergic bronchopulmonary aspergillosis

A 68-year-old woman with bronchial asthma presented to our outpatient clinic with a persistent cough, shortness of breath on exertion, and nocturnal insomnia due to dyspnea. Chest auscultation revealed marked expiratory wheezing. Peripheral blood tests revealed a white blood cell count of 9080/μL (normal range 3300-8600/μL), eosinophil count of 1616/μL (17.8%) (normal range 0-800/μL, 0-7.0%), serum total IgE of 16248 IU/mL (normal range <170 IU/mL), Aspergillus-specific IgE of 244 IUA/mL (normal range 0.10 IUA/mL), and Aspergillus precipitating antibody was positive. Pulmonary function tests could not be performed due to hospital regulations during the coronavirus disease 2019 (COVID-19) outbreak. Chest computed tomography showed bronchial wall thickening, mucus plugs, consolidation in both lungs, and high-attenuation mucus (Figure 1). Based on the diagnostic criteria of the Japanese Society of Allergy and Respiratory Medicine, a diagnosis of ABPA was made [2].

**Figure 2:**
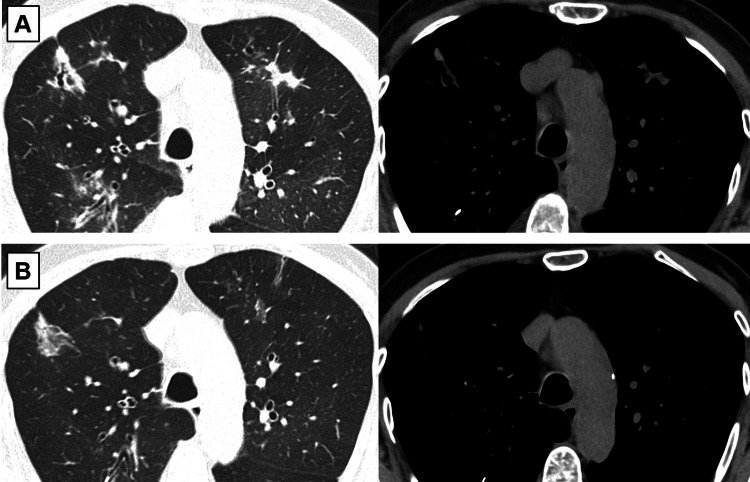
Computed tomography images of the chest (A) during symptom flare-ups two months after the initiation of benralizumab therapy and (B) when imaging findings markedly improved after the administration of dupilumab

The patient was initially treated with fluticasone/formoterol 1000/40 μg/day inhalation, followed by the addition of tiotropium 5 μg/day inhalation and oral itraconazole 400 mg/day. She had received inhalation instructions from the pharmacist and had no problems with the technique. Also, she had no fever, and the sputum examination was negative for infectious pneumonia with no significant bacterial growth. In spite of the need for additional medication to control her disease, she refused systemic corticosteroids due to concerns about side effects. Moreover, the patient refused to continue oral itraconazole because of her desire to avoid polypharmacy. Though a newer treatment, benralizumab, was introduced and provided temporary relief, two months later, she experienced symptom flare-ups, and chest imaging showed worsened findings (Figure 2A). After dupilumab was started as a new treatment regimen, both symptoms and imaging findings improved markedly (Figure 2B).

## Discussion

ABPA is a disease that arises due to host hypersensitivity to Aspergillus antigens, resulting in conditions such as poorly controlled asthma, mucous plug formation, and central bronchiectasis [1]. In the pathogenesis of ABPA, Th2 CD4+ T cells produce interleukins (ILs), and IL-5-mediated eosinophilic inflammation is considered particularly significant. IL-13 is also reported to be involved in the pathogenesis, especially with regard to mucus plug formation, as it has been reported to upregulate MUC5AC gene expression, resulting in increased mucin production and release by goblet cells of airway epithelial glands [3].

The fundamental treatment strategy for ABPA is systemic corticosteroids and antifungal agents [4-5]. However, in many cases, these drugs cannot be used for various reasons or are not effective enough. In particular, the side effects of long-term steroid use are often a clinical problem. In this context, it is noteworthy that the use of biological agents not only provides clinical and functional improvement but also offers a high potential for reducing or discontinuing systemic steroids [6]. Each of these biological agents targets the specific molecules associated with asthma exacerbations: e.g., benralizumab is an anti-IL-5 receptor alpha (IL-5Rα) monoclonal antibody, and dupilumab targets IL-4Rα, a common subunit of receptors for IL-4 and IL-13, which inhibits the signaling of both cytokines.

Although past case reports suggest that biological agents are effective in treating ABPA, their specific use remains a controversial issue. Mümmler et al. reported a case of ABPA in which benralizumab reduced eosinophil counts but did not alleviate symptoms or reduce the needed oral corticosteroid dose [7]. On the other hand, dupilumab is reported to bring complete symptom resolution, discontinuation of oral corticosteroids, and normalization of lung function. Mikura et al. experienced a case of relapse during steroid tapering after introducing mepolizumab, an anti-IL-5 monoclonal antibody, in a steroid-dependent ABPA patient [8]. They switched mepolizumab to dupilumab and were able to discontinue steroids without subsequent exacerbation of clinical symptoms. Ramonell et al. reported in their case series on the risk of eosinophilia that dupilumab is an effective treatment modality for ABPA [9]. In this case, the eosinophilia improved with oral corticosteroids. These reports suggest that dupilumab may be more effective against ABPA when mucus retention is present along with eosinophilic inflammation. The efficacy of dupilumab observed in the present case is consistent with previous reports. More extensive studies are needed to reach a firm conclusion about the expected therapeutic effect of dupilumab.

## Conclusions

We presented a case of ABPA well controlled with dupilumab after treatment failure with benralizumab. Although there are no clear criteria for the use of biological agents against ABPA, dupilumab’s ability to suppress IL-13 signaling makes it a good treatment option in cases where mucous plugs are especially problematic. This case also suggests that biological agents may be a promising treatment modality for cases in which corticosteroids cannot be used.

## References

[1] Agarwal R, Chakrabarti A, Shah A (2013). Allergic bronchopulmonary aspergillosis: review of literature and proposal of new diagnostic and classification criteria. Clin Exp Allergy.

[2] Asano K, Hebisawa A, Ishiguro T (2021). New clinical diagnostic criteria for allergic bronchopulmonary aspergillosis/mycosis and its validation. J Allergy Clin Immunol.

[3] Dunican EM, Watchorn DC, Fahy JV (2018). Autopsy and imaging studies of mucus in asthma. Lessons learned about disease mechanisms and the role of mucus in airflow obstruction. Ann Am Thorac Soc.

[4] Agarwal R, Muthu V, Sehgal IS (2022). A randomised trial of prednisolone versus prednisolone and itraconazole in acute-stage allergic bronchopulmonary aspergillosis complicating asthma. Eur Respir J.

[5] Agarwal R, Dhooria S, Singh Sehgal I (2018). A randomized trial of itraconazole vs prednisolone in acute-stage allergic bronchopulmonary aspergillosis complicating asthma. Chest.

[6] Albogami S (2021). Use of biologic drugs for treatment of allergic bronchopulmonary aspergillosis. Int J Pulm Respir Sci.

[7] Mümmler C, Kemmerich B, Behr J, Kneidinger N, Milger K (2020). Differential response to biologics in a patient with severe asthma and ABPA: a role for dupilumab?. Allergy Asthma Clin Immunol.

[8] Mikura S, Saraya T, Yoshida Y (2021). Successful treatment of mepolizumab- and prednisolone-resistant allergic bronchopulmonary aspergillosis with dupilumab. Intern Med.

[9] Ramonell RP, Lee FE, Swenson C, Kuruvilla M (2020). Dupilumab treatment for allergic bronchopulmonary aspergillosis: a case series. J Allergy Clin Immunol Pract.

